# Retroversion of the Uterus

**Published:** 1892-09

**Authors:** W. J. Matthews

**Affiliations:** Austin


For Daniel's Texas Medical Journal.

### RETROVERSION OF UTERUS.

W. J. MATTHEWS, M. D.

Read at Austin District Medical Association.

RETROVERSION of the uterus is so often associated with retroflexion and confounded with it, that I have deemed it proper in this paper to give a few definitions of both conditions from well known and recognized authorities. Tait defines retroversion as a condition in which the uterus is turned backward out of its proper axis, without its tube being at all bent or at least not greatly bent, and that retroflexion is a bending backward of the tube upon itself with more or less of a sharp curvature. Thomas states that retroversion is a posterior inclination of the uterus, so that the fundus approaches the sacrum and the cervix advances towards the symphisis pubis. Retroflexion is said to exist when the body of the uterus is bent toward the sacrum, so as to create an angle on its posterior wall. The last, and I think best definition, which I shall quote, is that of Harrison, viz : "Retroversion may be defined as the permanent dislocation backward of the fundus uteri, when the form of the uterus is such that axis of body and axis of cervix are identical. Retroflexion denotes the permanent backward dislocation of the fundus uteri with simultaneous flexion of the uterus over the posterior surface." Thus clearly understanding the meaning of these terus, we will now endeavour to investigate the cause of these backward dislocations of the uterus. It cannot be denied that retroversion and retroflexion often occur in those who have never borne children, and may be caused by violent blows on the abdomen, lifting heavy weights, or any force which produces increased intra-abdominal pressure.

It is asserted that such an accident can only happen when the bladder is distended and the uterus in a state of physiological retroversion. But as we are seldom called upon to treat uterine displacements in the virgin or unmarried, I will confine my remarks in this short paper to a discussion of backward displacement, the result of puerperal conditions, and first we would give a cause, the loss of support from morbid states of the uterine ligaments, especially the round ligaments. It is well known that the round ligaments are important aids in keeping the uterus in its normal position, and it is also evident that any condition which will cause relaxation of these ligaments, will favor retroversion of that organ. These ligaments, being composed of muscular tissue, similar to the uterus, it is reasonable to infer that any arrest of retrograde metamorphosis of that organ may also affect them, leaving them relaxed and less powerful than natural. Scahoni states that hypertrophy of the round ligaments constantly accompanies a natural pregnancy, and that when involution of the uterus is delayed, these ligaments are relapsed and are no longer able to perform their functions in retaining the uterus in normal position. Besides, in subinvolution all the uterine ligaments are weakened, favoring prolapse of the organ, which is but a step in the process of retroversion. Again, the almost universal practice of compelling women after their confinement to maintain the dorsal decubitus for one or two weeks, wearing all this time a tight abdominal binder are fruitful sources of these displacements.

Subinvolution itself is a cause of retroversion. Women leaving their bed and engaging in housework before complete involution has taken place, will often cause these displacements. Pelvic peritonitis, by producing cicatricial bands, can bind the uterus down in pouch of Douglas. Deep laceration of the cervix is given by Skeene as a cause. Such lacerations may extend into the parametrium, causing sufficient cicatricial contraction to dislocate the uterus. But of all the causes enumerated, perhaps the most frequent is prolapse of vagina from subinvolution and ruptured perineum.

The pathology of backward displacements remains an unsolved problem, some authorities holding that these malpositions affect injuriously the circulation and nutrition of the uterus, while others, equally eminent, assert that the alveolar hyperplasiæ so constantly found in these cases cause the displacement. The fact that subinvolution very often precedes retroversion would seem to favor the latter view.

The symptoms of backward displacement are numerous and misleading. I may mention the following: Severe backache, rectal tenesmus, dyspareunial menorrhagia, dysmenorrhœa, uterine affections.

Diagnosis.—The cervix is generally found in the axis of the vagina, low down in the pelvis, and easily reached, looking more forward than usual. Passing the finger behind the cervix into Douglas' cul de sac, we can usually feel the fundus in a false position; a rectal examination will be still more satisfactory. Lastly, resorting to the bimanual method and the use of the sound, our diagnosis would be complete.

Differential Diagnosis.—We have to differentiate retroversion and retroflexion from fibroid tumors on posterior wall of uterus, feces in rectum, prolapsed ovary, a small ovarian tumor, and I may mention here that a displaced kidney has been removed from this region by so great a man as Munde, and the diagnosis was not made until the operation was completed. But we will now proceed to the treatment of these displacements.

We must all recognize the fact that a woman may have a retroverted or retroflexed uterus, and yet remain in perfect health, without a single symptom referable to either condition. Such a case, of course, requires no treatment, but such cases are comparatively rare. There are two great classes of posterior displacement, which we require to keep constantly in mind in practice:

1. Those displacements where the uterus is freely moveable, with perhaps some inflammatory exudation, producing considerable tenderness on pressure, and when complete restoration of the organ to its normal position can be effected.

2. Those displacements in which the uterus is permanently bound down by cicatricial bands, the result of pelvic peritonitis or perhaps cellulitis.

But we would first briefly review the preventive treatment of these accidents.

Preventive medicine is the crowning glory of our art, and nowhere in this direction have more splendid victories been won than in the domain of gynecology. Much can be done to prevent these displacements, by the proper management of every case of labor. Antiseptic principles should be applied as thoroughly as possible in every case. After delivery, women should be cautioned against maintaining too rigidly the dorsal decubitus. She should be instructed to empty the bladder frequently, to avoid the use of binders or even corsets for a considerable time after confinement, to use suspenders to support her clothing. Laceration of the cervix should be repaired in due time. Perineal rents should be sutured immediately after labor. Constipation of bowels should be carefully guarded against, as this produces a dilatation of the rectum, where hard fecal masses accumulate, which press the cervix uteri toward the symphysis pubis, keeping the utero-sacral ligaments constantly in a state of tension, and thus favoring retroversion of the fundus uteri. In fact, everything should be done that would promote a speedy and perfect involution of a woman's reproductive organs.

This brings us to the curative treatment of the first class of cases, where no adhesions exist, and the retroverted uterus can be replaced, and first we must notice the various methods now employed in replacing this organ. The simplest is the manual method. The patient is placed in Sims' position, and the index and middle finger of the right hand are introduced into the vagina, palmar surface toward rectum. The body of the uterus is lifted on the tips of the finger until it becomes erect, then their dorsal surfaces are made to push this organ over into position. As the uterus becomes elevated, the middle finger is still kept in the post uterine space, to maintain what is gained, while the index finger is carried in front of the cervix, and this part is by pressure forced back toward the sacrum. The middle finger is now likewise placed in front of the cervix, and by both fingers this part is *forced toward* the sacrum and kept there for a short time.

Thomas claims the above method as superior to any other. Another method is to to place the woman in the knee chest position, and with the index or middle finger high up in the vagina, elevate or antivert the uterus. Others again advise, while the patient is in this position, to pull the cervix toward the hollow of sacrum with a Vulsellum forceps, and with two fingers in the rectum, correct the displacement. The genu-pectoral position of Campbell, as a remedial measure, is of doubtful utility. Hart and Barber state that the retroverted organ is never replaced by this method. Then there is an endless variety of uterine repositors a few of which are ingenious and useful instruments. Sponge probangs may at any time be useful, but each one will have his own favorite method, and we will now discuss the general management of these cases. In the large bulk of patients presenting themselves for treatment, the slightest pressure of the uterus with the examining finger causes such excruciating pain that preparatory treatment is necessary before any pessary could be worn. The vagina should be injected with hot water two or three times a day. Boro-glyceride tampons should be used, not only to deplete the uterus, but to keep it in position after manual replacement. This treatment should be continued until all tenderness around the uterus is removed and a hard rubber pessary can be worn with comfort, no properly adjusted pessary should cause pain. Indeed we may rest assured that when a pessary causes pain we are injuring our patient. The uterus should always be properly replaced before the introduction of any pessary is a rule which can never be violated.

You are all familiar with the various retroversion pessaries now in use and I will therefore not describe them. There are to-day many eminent gynecologists who do not believe in the mechanical theory of uturine displacements. They do not believe that the dislocation of the uterus is responsible for all the morbid conditions so often found in these cases. Many believe that congestion and inflammation of the uterus are the primary disturbances, and the displacement secondary. Tait, of England, and Gill Wylie, of New York, entertain views very much at variance with those who adhere strictly to the mechanical theory of displacements, and I will give their views almost in their own language. Tait writes thus: "I am sure that many women go about in this abnormal state without any symptoms at all. It is when subinvolution and retroversion coincide, still more when they go on to chronic metritis that trouble ensues. Then we have profuse and too frequent menstruation, leucorrhœa, backache and bearing down, etc. In such cases the uterus is easily replaced and pessaries might be used, but I always try to cure my patients first with chlorate of potash and ergot, and I succeed in 90 per cent., and finally say I hate pessaries, and I never use them if I can help it." He firmly believes that if you cure the endometritis and subinvolution so often found complicating these cases, the displaced uterus will resume it normal position.

Professor Wylie writes as follows: "That in many cases to-day being treated by the use of pessaries, and called cases of retroversion and retroflexion all symptoms can be permanently cured in a few weeks, by the use of the dilator, the drainage plug, sharp curette and simple intra-uterine applications properly made. Prof. Wm. M. Polk, of Bellvue Hospital, has also accomplished wonderful results in the cases through drainage of the uterus with sterilized gauze, although he is not opposed to the use of pessaries. I have always had some faith in pessaries, and yet so few of us possess the mechanical talent so necessary to fit and adjust pessaries, that the majority of us can not hope to have success in their use. When a brilliant and accomplished gynecologist like Tait says that he hates pessaries, and never uses them unless he can't help it, and Futeck, at the last meeting of the German gynecological society held in Halle, declared that he considered it easier to perform a laparotomy than to apply a well-fitting pessary, I must confess that my faith in pessaries has not been strengthened. Lately in a few cases, where the vagina was united to the cervix so near its lowest point as to leave no posterior pouch where a pessary could be used, I have resorted to uterine drainage using either Gill Wylie's plugs, or packing the uterus with sterilyzed gauze, using Dr. Polk's cervical speculum and Sims' tampon screw, and I can state that for so far I have had most gratifying results."

As a last resort, Alexander's operation for shortening the round ligaments might be tried which is a comparatively safe and successful one. But this brings us to the treatment of the second class of displacements, complicated with adhesions. Besides employing the measures already described, the vaginal vault should be frequently painted with tincture of iodine. Blisters should be applied over uterus. The simplest and safest methods for breaking up adhesions and liberating the uterus should be first tried. Massage is an efficient method, and if performed carefully is harmless. The next simplest method is Schultz's forcible replacement of uterus. The woman is placed in the lithotomy position, the rectum is well washed out with copious injections of hot water, the bladder is emptied, and an anæsthetic is administered. The index and middle finger of left hand are introduced high up in the rectum, and the position of uterus tubes and ovaries mapped out. The right hand as in the bimanual method, manipulating through the abdominal coverings, seizes the fundus and endeavors to bring it forward. While thus moving the uterus, the adhesions are made tense and easily felt by the finger in rectum, with which they are separated and broken up. In liberating the uterus we must be careful not to use too much force.

In difficult cases the same author, after dilating the cervix uteri sufficiently to introduce the index finger up to the fundus and forcibly bends the uterus in to proper position. Simple as these measures seem they are nevertheless not devoid of danger. As after treatment Schultz advises the use of ice-bags over the suprapubic region for twenty-four hours, and rest in bed for one week at least. In New York city a laparotomy is frequently performed to correct these version, when the adhesions cannot be broken up by the above method, and if the uterine appendages are diseased, they are removed at the same time. Prof. W. M. Polk, of Bellvue hospital, has done good work in this direction. After opening the abdomen and separating all adhesions, he inserts a glass drainage tube behind the uterus, and depends on it alone to keep the uterus in place. In the *American Journal of Obstetrics*, for June, 1887, he reports quite a number of cases cured by this operation. Lastly, there is the operation now termed ventro-fixation. The abdomen is opened in the median line as for ovariotomy, all adhesions binding the uterus down are broken up—the fundus brought forward and the uterus stitched with hardened cat-gut to the anterior abdominal wall, the suture being passed into the uterus at the insertion of the round ligament. These are grave surgical operations and the general practitioner would do well to give the simpler and safer method a faithful and patient conscientious trial. The two last described operations should only be undertaken by those who have had a large experience in pelvic or abdominal surgery. Be it ever remembered that though the displacement may be the cause of much suffering, yet they seldom shorten life. Millions of women suffering with these displacements long before the dawn of pelvic surgery, have bravely and nobly met the responsibilities of life, living to a good old age, and have gone down to their honored graves without their bodies being mutilated by a gynecological expert.

